# Reading between the Lines: RNA-seq Data Mining Reveals the Alternative Message of the Rice Leaf Transcriptome in Response to Heat Stress

**DOI:** 10.3390/plants10081647

**Published:** 2021-08-11

**Authors:** Charles Barros Vitoriano, Cristiane Paula Gomes Calixto

**Affiliations:** Department of Botany, Institute of Biosciences, University of São Paulo, Sao Paulo 05508-090, SP, Brazil; eubiologo@usp.br

**Keywords:** alternative splicing, thermotolerance, *Oryza sativa* L., plant, post-transcriptional regulation, meta-analysis

## Abstract

Rice (*Oryza sativa* L.) is a major food crop but heat stress affects its yield and grain quality. To identify mechanistic solutions to improve rice yield under rising temperatures, molecular responses of thermotolerance must be understood. Transcriptional and post-transcriptional controls are involved in a wide range of plant environmental responses. Alternative splicing (AS), in particular, is a widespread mechanism impacting the stress defence in plants but it has been completely overlooked in rice genome-wide heat stress studies. In this context, we carried out a robust data mining of publicly available RNA-seq datasets to investigate the extension of heat-induced AS in rice leaves. For this, datasets of interest were subjected to filtering and quality control, followed by accurate transcript-specific quantifications. Powerful differential gene expression (DE) and differential AS (DAS) identified 17,143 and 2162 heat response genes, respectively, many of which are novel. Detailed analysis of DAS genes coding for key regulators of gene expression suggests that AS helps shape transcriptome and proteome diversity in response to heat. The knowledge resulting from this study confirmed a widespread transcriptional and post-transcriptional response to heat stress in plants, and it provided novel candidates for rapidly advancing rice breeding in response to climate change.

## 1. Introduction

Global food security is being threatened by the rapid population growth in a changing environment. One of the most devastating factors of climate change for agriculture is the increase in average surface air temperatures, which not only causes a reduction in yield but also compromises grain quality [1,2]. Rice (*Oryza sativa* L.) is particularly susceptible to heat stress [3]. It is one of the most important crops in the world and it plays a major role in the human diet, accounting for 9% of the global crop production [4]. Heat stress is detrimental during both rice vegetative and, mostly, reproductive phases [3,5]. Every 1 °C rise in the minimum growing temperature reduces yield by at least 10%, also resulting in damaged/chalky grains [6,7]. Nevertheless, many rice-producing regions are in agricultural land predicted to face increases in average temperature of between 2.0 to 4.9 °C by 2100, posing a significant threat to rice production [8,9]. To react and adapt to the threat of ever-increasing average temperatures, society needs to invest in new rice cultivars that maintain high productivity despite these arduous environments [10]. From a crop improvement perspective, it is possible to develop increasingly heat-resistant rice cultivars. For this, it is important to understand the responses that allow rice to tolerate heat [11].

Plants depend on the perception and appropriate response to temperature for their survival. The thermal perception system(s) responsible for transducing temperature information to the plant cellular processes is one of the great unknowns in plant science. Two events have been hypothesised as the primary temperature sensing events: alteration of membrane fluidity and elevation of cytosolic free calcium concentration ([Ca^2+^]_cyt_) [12,13]. These are probably associated and are the earliest physiological events that occur upon temperature variation. Some responses to temperature may also be initiated by the direct physical effects of temperature on protein/nucleotide conformation and enzyme activity [14]. Sensing and signal transduction of non-stressful ambient temperature changes are somewhat different to sensing extreme temperature changes (temperature stress) [13]. Upon extreme temperature perception, plants respond through many genetic and physiological processes such as massive transcriptional changes, increases in levels of heat shock proteins, alterations in membrane composition and regulation of sugar, and changes in the rate of photosynthesis and respiration [12,13,15,16,17]. These responses are a major attempt to ensure plant survival by minimising the heat-stress damage, controlling leaf temperature, and developing a functional plasticity known as ‘temperature acclimation’ [12,13,15,18]. Essentially, leaves play an important role in adaptive strategies to overcome heat stress. For example, several studies in rice leaves observed differential expression of transcripts and proteins of dozens of genes upon heat shock [19,20,21,22,23]. Shi et al. [23] also demonstrated that high night temperature treatment did not cause any loss in rice spikelet fertility, whereas it caused a significant decline in overall plant biomass that ultimately resulted in yield and grain quality losses. Additionally, it has been suggested that high night temperatures are more harmful to grain weight in rice and other crops than high daytime temperatures because a deficiency of carbohydrates occurs in the leaves and culms due to increased respiration loss under high night temperatures [17]. Another study observed that if the day temperatures are high beyond the critical levels during flowering, flag leaf photosynthesis decreases substantially, resulting in disturbed source-sink assimilate transport [7]. Therefore, attempts to understand the molecular responses of rice leaves under rising temperatures merits considerable attention.

Molecular responses during environmental stresses have been mostly studied at the transcriptional level but post-transcriptional regulation and, in particular, alternative splicing (AS), is also an important mechanism involved in heat stress response. AS is where different splice sites are selected among precursor messenger RNAs (pre-mRNAs) of the same gene to produce different mRNAs, which can control gene expression and impact protein production and complexity [24,25]. AS is often overlooked in plant gene regulation studies, even though the biological importance of AS has been demonstrated in abiotic and biotic stress responses, as well as growth and development, light-dependent functions, and flowering [26,27,28,29,30,31,32,33,34]. AS occurs in more than 48–60% of intron-containing genes of many plant species, including rice [35,36,37]. Specifically, there are single-gene studies that have described heat-induced AS in rice [38,39,40,41,42,43]. Therefore, it is becoming increasingly apparent that post-transcriptional processing is also crucial to shape the plant transcriptome [30,44]. In particular, the exceptional versatility of regulation via AS is likely to play a prominent role in fine-tuning plant adaptation and acclimation to stress, holding great promise to expand the number of trait-related genes for molecular breeding [28,30,45,46].

Genomics and transcriptomics tools provide an advanced opportunity to uncover the unknowns of the genetic potential of plants responding to a range of environments [47]. High-throughput RNA-sequencing technology (RNA-seq) provides the most cost-effective and highly accurate means to address plant transcriptome diversity and dynamics in response to stresses [26,27,35,48,49,50]. RNA-seq data generate information on transcript variation in the genes, giving an opportunity to examine specific genes in detail to identify stress-dependent transcriptional and post-transcriptional regulation and, therefore, candidate genes for stress responses. Crucially, genome-wide studies have concluded that heat stress strongly affects AS patterns in several plant species, including *Arabidopsis thaliana*, *Triticum aestivum* (bread wheat), and *Physcomitrella patens* [48,51,52,53,54,55,56,57]. Part of the molecular mechanisms of the heat stress response is likely conserved among plants (*sensu stricto*) [58], and Chang et al. [51] have also observed that some of the heat-sensitive AS events are conserved, suggesting that the AS regulation has also been an advantage to plants’ survival very early in their evolution. It has been revealed that AS functions as a novel component of temperature memory, allowing plants to survive subsequent and otherwise lethal heat stress conditions [54,59]. Therefore, it has become clear that heat stress impacts splicing regulation and, consequently, the transcriptome [52,60,61]. However, transcriptome studies analysing heat stress in rice have completely overlooked at AS. Therefore, the present work aimed at carrying out an RNA-seq meta-analysis to identify AS regulation in response to high-temperature stress in rice leaves.

## 2. Results

### 2.1. Two-Thirds of the Rice Transcriptome Is Affected by Heat Stress at the Transcriptional and Post-Transcriptional Levels

To explore RNA-seq studies that contain data on rice leaf transcriptome in response to heat stress, we searched the Sequencing Read Archive (SRA) repository, available at the National Centre for Biotechnology Information (NCBI) database. This search returned 5 SRA studies of interest (Table 1). The experimental designs and sequencing strategies of each study follow the needs of the original studies and, therefore, differ in several aspects such as cultivar and read-length (Table 1 and Supplementary Table S1). Heat stress treatment, in particular, varied in terms of heat intensity (from 6 °C to 20 °C increase) and duration of stress (from 10 min to 12 days). To ensure that heat stress response was the only factor being tested in our study, a subset of RNA-seq datasets were extracted from these 5 SRA studies (SRP071314, SRP101342, SRP190858, SRP110860 and SRP065945) and are hereinafter referred to as experiments 1 to 5, respectively (Table 1 and Supplementary Table S2; more details in the Methods section).

After download and pre-processing of the RNA-seq datasets of interest, transcript-specific expression data was generated using Salmon [63] and the rice reference transcriptome available at the MSU Rice Genome Annotation Project Database [64]. This transcriptome contains transcripts from 55,548 genes, of which 6463 have two or more transcript isoforms (Supplementary Figure S1), allowing for AS studies in rice.

**Figure 1:**
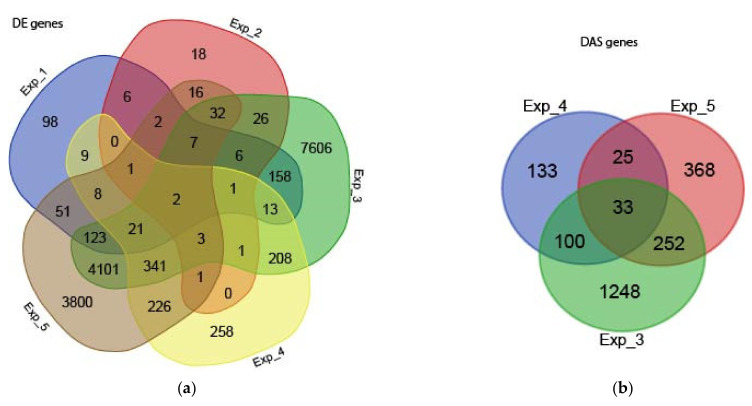
Venn diagram showing the overlap of (**a**) DE and (**b**) DAS genes from each experiment analysed here.

Differential gene expression and differential AS analysis were carried out with 3D RNA-seq [65] on an experiment-by-experiment basis, where we ensured that heat was the only factor being tested in our study. First, low expressed genes and transcripts were removed and expression data were normalised across samples. This identified a total of 26,834 genes expressed in at least one of the five heat stress experiments (Table 2), of which 60.6% were expressed in all experiments (Supplementary Figure S2A). The large overlap of expressed genes among the experiments confirms not only the similarity of the leaf/shoot transcriptomes from 2- to 5-week-old rice plants but also the power of the RNA-seq technology and analysis methods in detecting gene expressions across different sequencing strategies. Next, contrast groups were set up to compare the equivalent time-points with or without heat stress to control for time-of-day variation in expression due to photoperiodic and circadian changes. Differential gene expression (DE) analysis identified 17,143 genes that were significantly differentially expressed in at least one of the five heat stress experiments when compared with control samples (Table 2 and Supplementary Table S3). Experiments 3 and 5 greatly contributed to the identification of DE genes. This is probably due to increased statistical power mainly resulting from the higher number of biological replicates in experiment 3, and the higher number of RNA-seq datasets analysed in experiment 5. On average, ~40% of DE genes were upregulated and ~60% downregulated (Supplementary Figure S3). Next, we studied the overlap between the sets of DE genes from each experiment. Figure 1A shows a very small overlap among all experiments, reflecting differences in experimental designs of the original studies, mainly the different heat stress treatments and cultivars. To detect differentially alternatively spliced (DAS) genes, the expression changes between individual transcripts were compared to the gene level between conditions. We identified differential alternative splicing in 2162 genes in at least one of the five heat stress experiments (Table 2 and Supplementary Table S3), of which 1,355 were also DE genes (regulated by both transcription and AS) in at least one of the five experiments and 807 genes that were not DE (regulated by AS only) in any of the five experiments. Experiment 3 showed the highest number of DAS genes, which were overrepresented for the GO terms ‘gene expression’ and ‘RNA processing’, among others (Supplementary Figure S4). The high number of DAS genes in Experiment 3 is most likely due to the high average read length (Supplementary Table S1), which increases quantification accuracy of transcript-specific expression, allowing for a more comprehensive investigation on AS [66]. Next, we studied the overlap between the sets of DAS genes. For figure clarity, experiments 1 and 2, which identified only 3 and 2 DAS genes, respectively, were not included. Figure 1B shows a small overlap among all experiments, reflecting differences in experimental designs of the original studies, as observed for the overlap of DE genes. In summary, of the 26,834 genes expressed in rice leaves/shoots, 66.9% exhibited significantly altered levels of differential gene and/ or transcript-level expression, suggesting a widespread transcriptional and post-transcriptional response to heat stresses.

### 2.2. Novel Putative Heat Stress Response Genes in Rice

**Figure 2:**
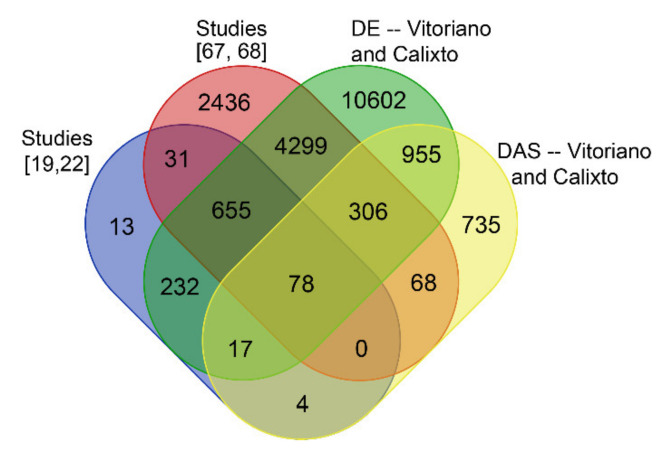
Venn diagram showing the overlap of DE genes from different studies and DE and DAS genes identified here.

The identification of DE and DAS genes raised the question of whether these have been described previously as heat-response genes. Of the five studies analysed here, only two have carried out a differential gene expression analysis that identified heat response genes [19,22] (Supplementary Table S4A). An additional two RNA-seq studies analysed the rice heat response while taking into account time-of-day variations in gene expression. Gonzáles-Schain et al. [67] have identified 630 DE genes in pollinated pistils and anthers of rice plants (cultivar N22) after 6 h at 38 °C, compared to 29 °C controls. Liu et al. [68] have identified 7573 DE genes in WT rice seedlings (cultivar Nipponbare) after 3 h at 45 °C, compared to 29 °C controls. Both studies have disclosed DE gene lists using MSU gene IDs (Supplementary Table S4A), which allowed a comparison with the sets of DE and DAS genes identified here. As expected, 99% of the heat response DE genes identified in Dossa et al. [19] and Wilkins et al. [22] were also identified here, while there was only a 69% overlap with DE genes identified by Gonzáles-Schain et al. [67] and Liu et al. [68] (Figure 2), reflecting differences in experimental designs among the studies, mainly heat stress treatment and tissue sample. Crucially, we identified an additional 10,602 novel DE-only heat stress-response genes (Supplementary Table S4B). 

The list of novel DE-only genes included 51 epigenetic proteins, 317 transcription factors, 27 heat-shock proteins, and 90 splicing-related factors (Supplementary Table S4C). The analysis here also identified 1,690 novel DAS heat stress-response genes (Figure 2). Therefore, in addition to the previously identified heat-response genes in rice, we provide a substantial contribution of novel genetic components underlying the response to temperature increase in rice leaves/shoots.

### 2.3. Key Regulators of Gene Expression Are Regulated by Heat-Induced AS

To understand the importance of heat-induced differential alternative splicing in rice, we asked if AS was potentially affecting the levels or function of key DAS genes. We focused our analysis on putative regulators of gene expression from four different categories: epigenetic proteins, transcription factors, heat-shock proteins and splicing-related factors. We observed heat-induced DAS in 9, 104, 15 and 45 genes from these categories, respectively (Table 3). Below we report important gene examples of each category and provide detailed evidence that heat stress-dependent AS is an important further layer of regulation that controls the function of genes related to the heat stress response in rice leaves.

#### 2.3.1. Example of DAS Genes Encoding Epigenetic Proteins

**Figure 3:**
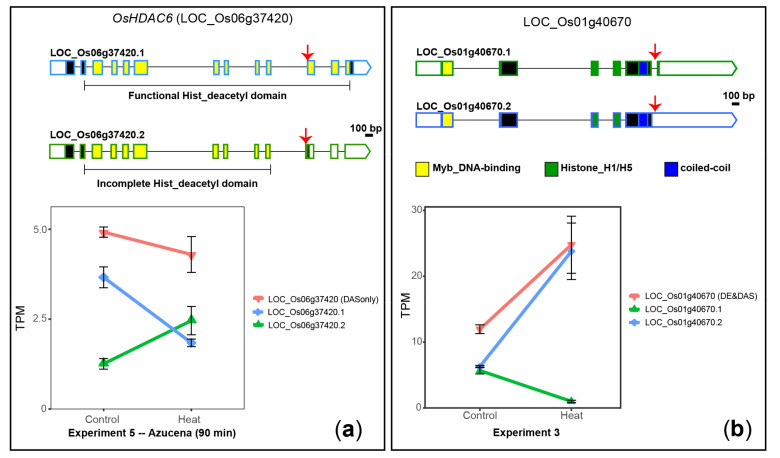
Heat-induced DAS of two genes encoding epigenetic proteins. (**a**) *OsHDAC6* (LOC_Os06g37420). (**b**) LOC_Os01g40670. 5′ and 3′ UTRs are open boxes; introns are represented with thin lines; coding sequences are shown as dark boxes, except for domain-encoding exons, which are coloured. Alternative splicing events are marked with red arrows. Error bars: standard error of the mean. TPM: Transcript per Million reads.

OsHDAC6 (LOC_Os06g37420), a novel DAS-only gene, is a histone deacetylase (H3-K14 specific) localised in chloroplasts [69] and it is involved in drought and salt stress responses [70]. Upon heat stress, *OsHDAC6* undergoes a rapid differential alternative splicing only (not DE) of two transcript isoforms that differ by an alternative 3′ splice site of exon 11 (Figure 3A). Isoform LOC_Os06g37420.1 codes for a protein containing the full histone deacetylase (Hist_deacetyl) domain (PFAM accession PF00850.12), whereas isoform LOC_Os06g37420.2 has a premature termination codon (PTC), which is probably recognised by the nonsense-mediated RNA decay (NMD) pathway, preventing its translation. The proportion of LOC_Os06g37420.1 transcripts decreased from 74% to 43% of the total transcripts (average ΔPS = 0.31) when rice plants were transferred from 30 °C to 40 °C for 90 min, respectively (Figure 3A). In summary, *OsHDAC6* expression is rapidly altered post-transcriptionally upon heat stress, and we speculate it might affect its protein levels, consequently impacting the epigenome.

LOC_Os01g40670, a novel DE&DAS gene, encodes a member of the Single Myb Histone (SMH) family protein, a plant-specific group of proteins involved in telomere structure and metabolism [71]. In control conditions, this gene produces two transcript isoforms, in equal proportions, that differ by removal or retention of the fifth intron (Figure 3B). The AS alters the C-terminal end of the predicted proteins, near a domain involved in stabilising protein dimer formation, known as the coiled-coil domain [71], suggesting the AS might affect protein function. We observed a significant up-regulation of this gene upon 24 h of heat stress (DE), where the proportion of intron-5-containing transcripts increased to 96% of the total transcripts (DAS; Figure 3B), which might impact telomere function.

#### 2.3.2. Example of DAS Genes Encoding Transcription Factors

**Figure 4:**
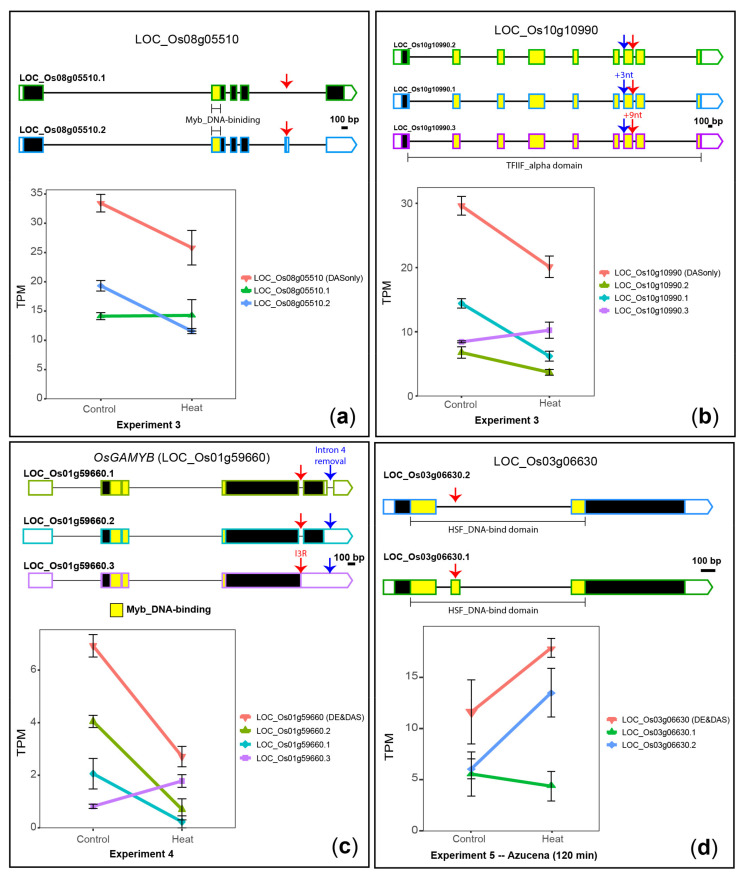
Heat-induced DAS of four genes encoding transcription factors. (**a**) LOC_Os08g05510. (**b**) LOC_Os10g10990. (**c**) *OsGAMYB* (LOC_Os01g59660). (**d**) LOC_Os03g06630. 5′ and 3′ UTRs are open boxes; introns are represented with thin lines; coding sequences are shown as dark boxes, except for domain-encoding exons, which are coloured. Pairs of alternative splicing events are marked with red or blue arrows, where labelling is added when the event is unique to the marked transcript when compared to the other transcripts. Error bars: standard error of the mean.

LOC_Os08g05510, a novel DAS-only gene, encodes a member of the MYB transcription factor family found in the nucleus [72]. This gene produces two transcript isoforms that differ by skipping or including the fifth exon (Figure 4A). The AS alters the C-terminal end of the predicted proteins, suggesting the AS might affect protein function. The relative abundance of these transcripts is reversed in response to 24 h of heat stress (Figure 4A), which suggests this gene is involved in the stress response of rice.

LOC_Os10g10990, a novel DAS-only gene, encodes a transcription initiation factor IIF alpha-subunit-domain-containing protein (TIFII-alpha). This gene produces three transcript isoforms that differ at alternative 5′ and 3′ splice sites of exon 7, which contains the coding sequence for the TFIIF_alpha domain (PFAM accession PF05793.5) (Figure 4B). As a result, AS can putatively affect the DNA binding function of LOC_Os10g10990, such as affecting the number of its DNA target sites. We observed an isoform switch event between transcripts LOC_Os10g10990.1 and LOC_Os10g10990.3 upon 30 min (Supplementary Figure S5A) and 24 h of heat stress (Figure 4B) when compared to controls, which suggests different targets are controlled by LOC_Os10g10990 in different temperature conditions.

*OsGAMYB* (LOC_Os01g59660), a novel DE&DAS gene, encodes a member of the MYB transcription factor family involved in gibberellins-regulated events, such as floral organ and pollen development [73]. In rice leaves/shoots, this gene produces three transcript isoforms that differ by removal or retention of the third and/or the fourth introns (Figure 4C). The I3R event shortens the C-terminal end of the predicted proteins when compared to the other isoforms, suggesting altered protein function. In control conditions, I3R isoforms represent only 12% of the total transcripts, while 10 min of heat stress increases its relative and absolute abundance to 66% of the total transcripts (average ΔPS = −0.54, Figure 4C), despite downregulation at the gene level, suggesting an involvement of *OsGAMYB* in early heat stress response of rice.

LOC_Os03g06630, a DE&DAS gene, is a heat stress transcription factor involved in the heat stress response of rice leaves, as identified previously [22,68] and confirmed here. This gene produces two transcript isoforms that either include or skip exon 2, which contains the coding sequence for the DNA binding domain HSF_DNA-bind (PFAM accession PF00447.10) (Figure 4D). As a result, AS can putatively either impair DNA binding function or increase the number of DNA target sites of LOC_Os03g06630. In control conditions, both isoforms are equally abundant, but upon heat stress, exon-2-skipping isoform (LOC_Os03g06630.2) increases both its relative and absolute abundances (Figure 4D), suggesting a differential control by LOC_Os03g06630 on downstream targets.

#### 2.3.3. Example of DAS Genes Encoding Heat-Shock-Related Proteins

**Figure 5:**
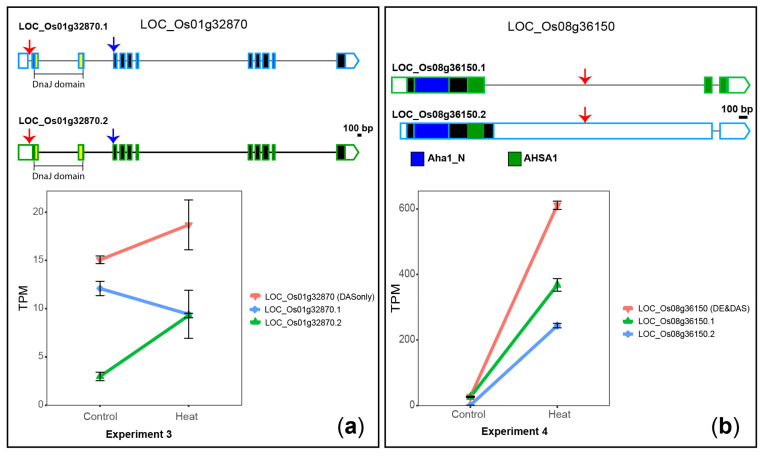
Heat-induced DAS of two genes encoding heat-shock-related proteins. (**a**) LOC_Os01g32870. (**b**) LOC_Os08g36150. 5′ and 3′ UTRs are open boxes; introns are represented with thin lines; coding sequences are shown as dark boxes, except for domain-encoding exons, which are coloured. Pairs of AS events are marked with red or blue arrows. Error bars: standard error of the mean.

LOC_Os01g32870, a novel DAS-only gene, is a heat shock protein containing a DnaJ domain and it is actively regulated at the protein level during rice embryogenesis [74]. This gene shows temperature-dependent DAS-only, altering the relative levels of two isoforms that differ by two AS events: (1) retention or removal of the first intron, which alters the 5′ untranslated region (UTR); and (2) an alternative 3′ splice site of exon 4, which adds or removes, respectively, 3 amino acid residues of the predicted proteins, near the DnaJ domain (PFAM accession PF00226.24) (Figure 5A). Post-transcriptional regulation by intron retention (IR) within the 5′-UTR can affect transport, stability and translatability of mRNAs [75], whereas changes in the protein primary structure can impact its function. These results suggest that AS of LOC_Os01g32870 in response to heat stress might impact its protein levels and function.

LOC_Os08g36150, a DE&DAS gene, encodes a member of the activator of the Hsp90 ATPase homolog 1-like family protein. Upon heat stress, this gene is upregulated at the gene level, as identified previously [22,68] and confirmed here. Our careful investigation at the transcript level demonstrates that LOC_Os08g36150 also undergoes a rapid heat-induced DAS. For example, in experiment 4, intron-1-retained (I1R) isoform LOC_Os09g31482.2 changes from 5% of the total transcripts in 22 °C control conditions up to 40% of the total transcripts after a 10-min-treatment at 42 °C (average ΔPS = −0.35) (Figure 5B). The roles of both the fully spliced (LOC_Os09g31482.1) and the I1R transcripts are unknown but we can propose three hypotheses. First, both isoforms are translated, generating proteins with a complete and an incomplete, respectively, AHSA1 domain (PFAM accession PF08327.4), required for full functionality. Second, the PTC of I1R transcripts is recognised by the NMD pathway and it is degraded, which prevents its translation. Lastly, the I1R transcript is retained in the nucleus, as observed for many intron-containing transcripts in animals and plants [76,77,78], where it can be post-transcriptionally spliced, generating the fully spliced isoform. Therefore, it is likely the heat-induced AS in LOC_Os08g36150 is an important mechanism that controls its protein levels.

#### 2.3.4. Example of DAS Genes Encoding Splicing-Related Factors

**Figure 6:**
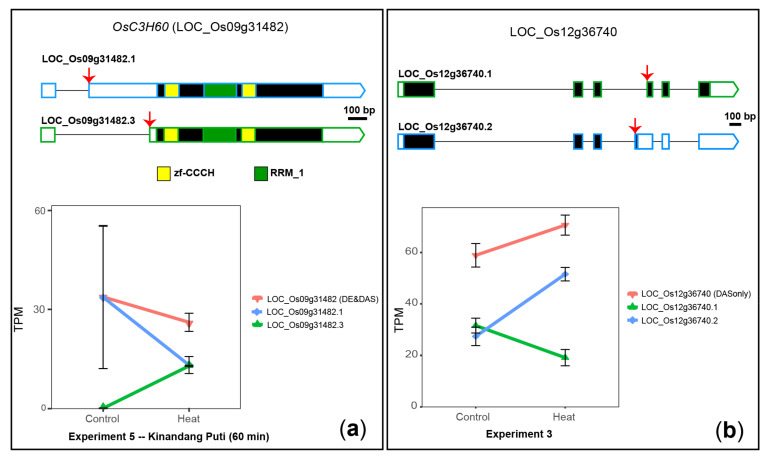
Heat-induced DAS of two genes encoding splicing-related factors. (**a**) *OsC3H60* (LOC_Os09g31482). (**b**) LOC_Os01g40670. 5′ and 3′ UTRs are open boxes; introns are represented with thin lines; coding sequences are shown as dark boxes, except for domain-encoding exons, which are coloured. AS events are marked with red arrows. Error bars: standard error of the mean.

*OsC3H60* (LOC_Os09g31482), a DE&DAS gene, encodes a U2 snRNP auxiliary factor small (35 kDa) subunit A and it is required in diverse biological roles by associating with RNA molecules [79]. In addition to differential gene expression due to heat stress, identified previously [22,68] and confirmed here, *OsC3H60* also undergoes significant heat-induced AS. For example, in experiment 3, isoform LOC_Os09g31482.1 changes from 50% of the total transcripts in 28 °C control conditions down to 11% after a 24 h-treatment at 45 °C (average ΔPS = 0.39) (Supplementary Figure S5B). In experiment 5, isoform LOC_Os09g31482.3 changes from <1% of the total transcripts in 30 °C control conditions up to 50% after 60 min at 40 °C (average ΔPS = −0.5) (Figure 6A). The two isoforms differ by an alternative 3′ splice site of exon 2 that alters the 5′ untranslated region (UTR) (Figure 6A), which can impact transcript stability and/or translatability, for instance adding/removing upstream ORFs [80], putatively controlling protein levels in a fast molecular response to temperature increase.

LOC_Os12g36740, a novel DAS-only gene, encodes a putative member of the serine/arginine-rich (SR) splicing factor family, a group of proteins involved in pre-mRNA splicing [81]. In control conditions, this gene produces two transcript isoforms, in almost equal proportions, that differ by an alternative 3′ splice site of exon 4, which either introduces or not a PTC that is likely recognised by the NMD pathway (Figure 6B). Therefore, LOC_Os12g36740 might produce only one protein isoform, from transcript LOC_Os12g36740.1. We observed that LOC_Os12g36740 undergoes a DAS change only (not DE) upon 24 h of heat stress, where the proportion of PTC-containing transcripts increased to 75% of the total transcripts (average ΔPS = −0.27) (Figure 6B), which likely results in decreased protein levels.

## 3. Discussion

We carried out a robust meta-analysis of publicly available RNA-seq datasets to explore the rice heat response and identified 15,818 DE-only genes and 2162 DAS genes. These results go in line with the widespread transcriptional and post-transcriptional response to heat stress observed in other plant species [48,51,52,53,54,55,56,57]. Notably, 10,602 DE-only and 1690 DAS genes were not identified in previous gene-level RNA-seq analyses [19,22,65,66] and are novel heat response genes, and included many key regulators of gene expression, namely heat-shock-related protein genes, transcription factors, epigenetic proteins and splicing-related factors, among others. Crucially, DAS of regulators of gene expression suggests further effects of AS at the epigenetic, transcriptional, post-transcriptional and post-translational levels.

Our detailed analysis on heat-induced AS of key regulators of gene expression suggests that the primary consequence and function of AS is to affect protein expression. We observe that this could be achieved qualitatively and/or quantitatively, as reported by other studies [24,25]. Regarding qualitative control, heat-induced AS likely increased the possible number of protein isoforms, which can have a different activity, localisation, stability and ability to interact with other proteins and substrates [25]. Concerning quantitative control, heat-induced AS could regulate protein expression levels by determining an mRNA cellular localisation, modifying how well an mRNA is translated, and/or decreasing mRNA stability and targeting it for degradation [25,82]. Such AS is especially crucial when altered protein levels are needed despite opposing transcriptional behaviour [83]. Therefore, the extensive AS information identified here demonstrates a much higher degree of complexity of gene regulation in rice in response to heat that has been significantly underestimated by analysis of RNA-seq data at the differential gene expression level only.

RNA-seq is a powerful tool to analyse the complex molecular mechanisms underlying plant response to environmental cues. It has the potential to detect genome-wide gene expression at the level of both genes and transcripts, providing a means to study post-transcriptional processes such as AS. Heat stress studies of rice plants using RNA-seq have only focused on differential gene expression analysis [19,22,67,68], overlooking at AS. This is partially explained by the fact that (1) accurate genome-wide quantification of transcript variants generated by AS and (2) the cost and complexity in analysing AS are neither trivial. We are now in a position where both of these issues have been solved and we can now readily unlock the discovery potential of underexplored publicly available RNA-seq data [65]. Here, we used Salmon [63] and the 3D RNA-seq [65] tools to analyse a subset of RNA-seq datasets extracted from 5 SRA studies. Validation of this pipeline—including choice of software, parameters and methods—for accurate quantification of transcript-specific abundances for AS analyses was demonstrated previously using data of the Arabidopsis cold-response [49,84]. Therefore, we are confident that our analysis allowed the generation of robust quantitative transcript-specific expression data and full differential expression and differential AS analyses, shedding light on the important problem of heat-induced AS in rice leaves.

We confirmed two important caveats on DAS analysis while carrying out our studies. Firstly, RNA-seq read length has a direct effect on the identification of DAS genes. Longer reads are generally better for (pseudo-)mapping because they are more likely to differentiate alternative transcripts, improving isoform-specific detection and quantification [66]. For example, experiment 3 analysed here had the highest average read length and it retrieved the highest number of DAS genes. Therefore, the longest reads (≥100 bp) should be used if alternative splicing analysis is the biological question one seeks to answer. Secondly, a high-quality reference transcript dataset (RTD) is crucial for a comprehensive alternative splicing study using the robust RNA-seq analysis carried out here [84,85]. The rice reference transcriptome we used [64] contains 6,463 genes that have two or more transcript isoforms. Although this dataset allowed the identification of DAS genes reported here, it is an underrepresentation of alternative splicing in rice genes [36]. Therefore, frequent improvements of the rice RTD holds the key if we are to continually expand our understanding of the regulations of gene expression in rice.

One final question that heat-sensitive AS raises is how variation in transcript isoforms is regulated by temperature changes. Temperature might have a direct effect on the rate of biochemical reactions that potentially affect transcription and splicing. Splicing is largely co-transcriptional and slower rates of RNA polymerase II elongation promote selection of alternative splice sites [86] and epigenetic modifications affect AS patterns in response to heat [87,88]. Additionally, heat is known thermodynamically to unfold mRNAs at AU-rich regions located in both 5′ and 3′ UTRs, which in turn facilitates RNA degradation [14]. Another possibility is that heat-affected transcript isoforms might involve miRNA regulation mechanisms [89]. Lastly, alternative pre-mRNA splicing is highly influenced by exonic and intronic regulatory cis-sequences as they serve as binding sites for trans-acting factors such as heterogeneous nuclear ribonucleoproteins and SR proteins, which then interact with the spliceosome [24]. The level and activity of hundreds of splicing factors change in response to temperature due to the transcriptional and post-transcriptional regulation, and the changes in cellular localisation and post-translational modifications, suggesting their involvement in thermal stress AS regulation [49,51,52,90,91,92]. In our work, we identified DE and/or DAS genes coding for epigenetic proteins and splicing-related factors, suggesting they are potential candidates involved in the differential AS regulation in response to heat stress in rice.

Novel molecular candidates involved in rice stress tolerance is widely recognised as critical. Despite the current knowledge on rice heat-tolerance [3,7], breeding approaches to develop heat tolerance in rice has had limitations [3,93] and challenging factors are currently bringing further pressure onto breeding better crops for the future [94]. Therefore, RNA-seq data mining and DAS analysis, such as the ones carried out here, greatly contribute to expanding our knowledge on the effect of environmental stresses in plants. In terms of future directions, molecular experiments might be used to further confirm our results and to explore this novel AS regulation in different HS conditions. Moreover, the construction of splicing and transcriptional networks from RNA-seq data on rice heat stress will further explain the roles of AS and transcriptional responses to thermotolerance, providing additional insight and strategies to rapidly advance rice breeding in response to changing environments.

## 4. Materials and Methods

### 4.1. Selection and Pre-Processing of RNA-seq Datasets

**Figure 7:**
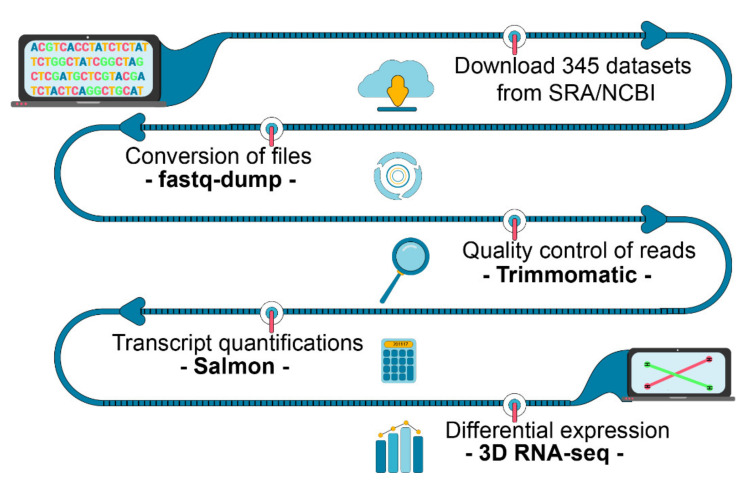
Flow chart of the main bioinformatics analysis carried out in our study.

The flow chart of the meta-analysis carried out here is shown in Figure 7. The RNA-seq studies analysed in this work have been described previously [14,19,20,22,60] and were selected from the SRA repository according to the following criteria: (1) studies should contain data from leaf samples of wild-type (non-genetically modified) rice plants for both control and heat stress treatments; (2) reads should have ≥ 50 bp, to increase the performance of transcriptome analysis focused on alternative splicing. For each SRA study identified, additional filtering was carried out to reflect the objectives of our study. Firstly, RNA-seq datasets containing data from abiotic stresses not related to heat, namely drought and salt stresses, were not included in our analysis. Additionally, in two of the studies [19,20], the heat stress treatment included pathogen- and mock-inoculated tissues. Only samples of healthy (mock-inoculated) tissues were analysed here. In the work of Luo et al. [62], the heat stress treatment was applied for 1, 3, 6, 12, and 24 h after the 0 h control. To take full account of the time-of-day variations in gene expression due to photoperiod [95] and circadian control [96], only time points 0 h (control) and 24 h (heat stress) were analysed. Aside from their temperature difference, time point 24 h is virtually identical to 0h control in terms of their zeitgeber, the only difference being they are 24 h apart, such that few time-of-day variations in transcript expression were expected when comparing these time points. Taking full account of the time-of-day in our analysis is especially important because the transcriptome dynamics of rice leaves is widely affected by the entrained circadian clock and temperature [97]. Lastly, in the work of Su et al. [14], RNA-seq libraries were generated from 14-day-old rice shoot tissue after a brief (10 min) treatment at 22 °C (control) or 42 °C (heat shock), which were the only datasets analysed here. The authors carried out additional RNA-seq experiments that quantified transcript abundance change over a longer time course post-heat shock, which they call it ‘recovery period’. In this recovery period, plants that have undergone 10 min of 42 °C or 22 °C treatments were transferred to 30 °C conditions for 10 min, 50 min, 1 h 50 min and 9 h 50 min. Neither of those additional RNA-seq datasets was analysed here because the temperature increase in control samples (from 22 °C to 30 °C) compared against temperature decrease in heat shock samples (from 42 °C to 30 °C) adds additional covariates in the heat stress response, which is not the interest of our work. In summary, 345/637 RNA-seq datasets were selected from 5 SRA studies (Supplementary Table S2), which are referred to as experiments 1 to 5 (Table 1).

Datasets were downloaded using an in-house script. First, fastq-dump, available in the SRA-Toolkit version 2.10.9 http://ncbi.github.io/sra-tools/ (accessed on 8 January 2021), was run with the options “--gzip” and “--split-spot”. Residual adaptor sequences at both 5′ and 3′ ends were removed from raw reads using Trimmomatic version 0.39 [98] with quality score threshold set at 33 and a minimum length of the trimmed read kept at 36.

### 4.2. Quantification of Transcript-Specific Expression

To deliver accurate quantitative transcript-specific expression data from 345 RNA-seq datasets (Supplementary Table S2) we used Salmon version 1.4.0 [63], with default parameters, and the rice reference transcriptome (all cDNAs) available at the MSU Rice Genome Annotation Project Database, Osa1 Release 7 [64].

### 4.3. Data-Exploratory Analysis with 3D RNA-seq

To perform a full differential gene expression and differential AS analysis for each study we used the 3D RNA-seq app [65]. The program’s pipeline can be divided into three modules: data generation, data pre-processing and 3D analysis.

For the data generation module, time (when applicable), cultivar (when applicable), treatment, and biological replicates were set as factors. Then, read count and Transcript per Million reads (TPM) information were generated from each Salmon output file using the lengthScaledTPM method.

For the data pre-processing module, low expressed genes and transcripts were removed. This removal was carried out independently for each study, where an expressed transcript should have a minimum count per million (CPM) of 1, in at least *m* samples. Each cut-off setting (*m*) was chosen based on its effect on the mean-variance trend for the RNA-seq log_2_-transformed read counts, which should follow the negative binomial distribution. Next, to reduce sequencing biases, normalisation of read counts was carried out across the libraries for each study using the weighted Trimmed Mean of M-values (TMM) method.

For the 3D analysis module, contrast groups of simple pair-wise analyses were defined, where corresponding time-points from the heat stress samples were compared to those of the control samples. For the work of Wilkins et al. [22], not only control samples but also ‘recovery from heat shock’ samples were compared, in parallel, to heat shock samples. This approach would allow for the identification of variations in gene expression due to the introduction and withdrawal of the heat stress. Next, we applied the limma voomWeights pipeline for differential expression analysis at both gene and transcript levels, setting the following thresholds: absolute log_2_-fold change (*L*2*FC*) ≥ 1, adjusted *p*-value (FDR) < 0.05, and absolute change in percentage spliced (ΔPS) ≥ 0.1 (F-test). The ΔPS is calculated as the percentage change in the abundance of a transcript compared to the total expression level of its gene.

### 4.4. Gene Functional Annotations

Venn diagrams were generated at http://bioinformatics.psb.ugent.be/webtools/Venn/ (accessed on 19 March 2021). Putative gene functions and gene lists were obtained from the MSU Rice Genome Annotation Project Database [64] and Oryzabase [99] on 25 March 2021. Schematic diagrams of gene structures were made with the help of the Exon–Intron Graphic Maker program http://wormweb.org/exonintron (accessed on 22 February 2021). Gene Ontology statistical overrepresentation test was carried out with Panther version 16.0 http://pantherdb.org/ (accessed on 8 April 2021), where we chose the annotations set ’GO biological process complete’ and the binomial test with no correction.

## Figures and Tables

**Table 1:** Characteristics of the experimental design for each study analysed here.

Experiment	SRA Study [Reference]	Cultivar(s)	Plant Age before Heat Stress	Temperature at Control	Heat Stress (Analysed Here)	Sample Tissue
1	SRP071314 [19]	IR24 and IRBB67	2-week-old	29 °C/21 °C day/night	35 °C/31 °C day/night for 7, 10 and 12 days	leaves
2	SRP101342 [20]	IRBB61	~4-week-old	29 °C/23 °C day/night	35 °C/29 °C day/night for 1 week	leaves
3	SRP190858 [62]	Nipponbare	2-week-old	28 °C	45 °C for	stem and leaves
4	SRP110860 [14]	Nipponbare	2-week-old	22 °C	42 °C for 10 min	shoot
5	SRP065945 [22]	Azucena, Kinandang Puti, Palawan and Tadukan	2-week-old	30 °C/20 °C day/night ^1^	40 °C (analysed every 15 min for up to 4.)	leaves (10 cm above the seed)

^1^ After the heat stress, some plants were returned to the 30 °C chamber (heat shock recovery).

**Table 2:** Numbers of genes in each category of heat stress responsiveness for each experiment.

	Experiment					
	1	2	3	4	5	Total ^3^
Expressed	20,633	19,702	22,373	20,728	21,024	26,834 ^4^
DE (DE-only)	506 (506)	122 (122)	12,650 (11,883)	1093 (1060)	8736 (8525)	17,173 ^4^ (15,818)
Not DE ^1^	20,127	19,580	9723	19,635	12,288	9661
DE + DAS ^1^	0	0	767	33	210	1355
DAS (DAS-only)	3 (3)	2 (2)	1633 (866)	291 (258)	678 (468)	2162 ^4^ (807)
No regulation ^1,2^	20,124	19,578	8857	19,377	11,610	8854

^1^ Number of genes is calculated column-wise. ^2^ Expressed gene that are not differentially expressed (DE) nor differentially alternatively spliced (DAS). ^3^ Each row contains a number of unique genes (no duplication). ^4^ Genes identified in at least one experiment.

**Table 3:** List of key regulators of gene expression regulated by heat-induced alternative splicing (AS) and their DAS statistics.

Category	Gene ID	Lowest Adjusted *p*-Value ^1^	ΔPS ^2^	Gene ID	Lowest Adjusted *p*-Value ^1^	ΔPS ^2^
Epigenetic proteins	LOC_Os01g40670	0	0.434	LOC_Os01g11960	0.001	0.627
	LOC_Os01g51154	0	0.435	LOC_Os06g37420	0.006	−0.393
	LOC_Os03g51230	0	−1	LOC_Os01g42630	0.027	0.168
	LOC_Os01g14370	0	0.240	LOC_Os05g04330	0.043	0.309
	LOC_Os02g07450	0	0.147			
Transcription factors Transcription factors	LOC_Os01g65080	0	0.2994	LOC_Os01g15460	0.0022	0.1113
	LOC_Os05g16660	0	0.7178	LOC_Os09g35920	0.0022	0.1088
	LOC_Os02g23823	0	0.6136	LOC_Os02g45480	0.0024	0.1875
	LOC_Os03g12370	0	0.6523	LOC_Os05g43380	0.0032	0.1025
	LOC_Os02g29550	0	0.1487	LOC_Os10g35300	0.0045	−0.3725
	LOC_Os02g49700	0	0.3627	LOC_Os03g08470	0.0051	0.1157
	LOC_Os02g06584	0	−0.2976	LOC_Os12g29520	0.0051	0.4068
	LOC_Os01g70270	0	0.1717	LOC_Os03g48970	0.0059	0.1186
	LOC_Os03g55590	0	−0.4274	LOC_Os11g32900	0.0059	−0.2112
	LOC_Os01g48060	0	−0.1346	LOC_Os02g36880	0.0059	0.1394
	LOC_Os10g10990	0	0.5090	LOC_Os09g26420	0.0062	−0.6059
	LOC_Os06g09390	0	0.1143	LOC_Os06g47150	0.0066	0.2087
	LOC_Os07g07350	0	0.1789	LOC_Os06g10880	0.0074	−0.4070
	LOC_Os02g53500	0	0.1322	LOC_Os09g32510	0.0082	0.1435
	LOC_Os02g05510	0	−0.2219	LOC_Os10g16440	0.0083	−0.1962
	LOC_Os10g41200	0	0.1377	LOC_Os07g44090	0.0083	−0.3019
	LOC_Os02g03340	0.0001	0.6246	LOC_Os04g57010	0.0085	0.2016
	LOC_Os03g13614	0.0001	0.3034	LOC_Os12g06200	0.0098	−0.1425
	LOC_Os08g44820	0.0001	0.3851	LOC_Os02g07780	0.0099	0.2247
	LOC_Os05g48870	0.0001	0.6216	LOC_Os04g49450	0.0104	0.3868
	LOC_Os05g35170	0.0001	0.1476	LOC_Os01g62410	0.0112	−0.4320
	LOC_Os01g56550	0.0001	0.2620	LOC_Os07g30130	0.0118	−0.2587
	LOC_Os03g48450	0.0002	−0.4427	LOC_Os03g02160	0.0122	0.1429
	LOC_Os06g21390	0.0002	0.2526	LOC_Os03g60130	0.0129	0.2355
	LOC_Os03g32590	0.0002	−0.1945	LOC_Os12g41880	0.0141	−0.1351
	LOC_Os08g07970	0.0002	−0.3772	LOC_Os06g47350	0.0146	0.1456
	LOC_Os03g52594	0.0002	−0.1957	LOC_Os02g07840	0.0152	0.2611
	LOC_Os08g37480	0.0002	0.1214	LOC_Os08g40900	0.0163	−0.1108
	LOC_Os03g44900	0.0002	0.2145	LOC_Os01g59660	0.0165	−0.5389
	LOC_Os10g25770	0.0003	0.1201	LOC_Os02g54120	0.0165	−0.1672
	LOC_Os04g50060	0.0003	0.3275	LOC_Os05g32270	0.0168	0.3684
	LOC_Os05g34830	0.0003	−0.5755	LOC_Os09g29830	0.0172	−0.7682
	LOC_Os07g04700	0.0003	0.4234	LOC_Os04g54474	0.0198	0.2022
	LOC_Os04g56640	0.0003	0.5046	LOC_Os05g37170	0.0204	0.2608
	LOC_Os07g43530	0.0003	0.5772	LOC_Os03g06630	0.0222	0.4669
	LOC_Os05g50930	0.0004	−0.1649	LOC_Os05g51150	0.0259	0.1038
	LOC_Os01g68860	0.0005	0.3098	LOC_Os07g48450	0.0260	0.6706
	LOC_Os07g41370	0.0005	0.1907	LOC_Os02g49840	0.0270	0.4198
	LOC_Os06g51260	0.0007	−0.2272	LOC_Os08g05510	0.0272	−0.1296
	LOC_Os04g53540	0.0009	0.5996	LOC_Os05g36160	0.0282	0.1058
	LOC_Os01g64310	0.0009	0.2160	LOC_Os10g31850	0.0283	0.1206
	LOC_Os11g16290	0.0011	0.2475	LOC_Os05g48500	0.0285	0.1742
	LOC_Os06g01230	0.0011	0.1860	LOC_Os04g47990	0.0287	0.1397
	LOC_Os01g24070	0.0013	0.3312	LOC_Os12g06850	0.0331	0.2045
	LOC_Os03g31230	0.0013	0.3120	LOC_Os03g52450	0.0339	0.1985
	LOC_Os02g34630	0.0013	−0.1344	LOC_Os06g04010	0.0359	0.6013
	LOC_Os12g13170	0.0015	0.1969	LOC_Os01g69910	0.0387	−0.2286
	LOC_Os03g07880	0.0016	−0.4076	LOC_Os01g13520	0.0426	−0.1300
	LOC_Os05g01710	0.0016	−0.3429	LOC_Os05g10620	0.0439	−0.5963
	LOC_Os07g41720	0.0017	0.2625	LOC_Os08g19650	0.0455	0.1413
	LOC_Os09g38790	0.0019	−0.2019	LOC_Os12g18120	0.0462	0.1521
	LOC_Os01g59350	0.0020	−0.3579	LOC_Os05g01020	0.0492	0.1114
Heat shock proteins	LOC_Os03g18200	0	−0.5586	LOC_Os03g60790	0.0013	−0.5030
	LOC_Os08g36150	0	0.3516	LOC_Os02g52270	0.0031	−0.3718
	LOC_Os01g33800	0	0.1277	LOC_Os04g24180	0.0042	−0.4988
	LOC_Os02g50760	0	0.1880	LOC_Os07g43870	0.0050	0.1630
	LOC_Os08g39140	0.0002	0.1568	LOC_Os06g06490	0.0064	−0.7906
	LOC_Os02g54130	0.0003	−0.4082	LOC_Os02g52150	0.0084	−0.1224
	LOC_Os03g27460	0.0003	0.4591	LOC_Os08g32130	0.0151	0.3409
	LOC_Os01g32870	0.0008	−0.2986			
Splicing-related factors Splicing-related factors	LOC_Os09g31482	0	0.7111	LOC_Os03g62640	0.0034	0.2330
	LOC_Os09g03610	0	0.1309	LOC_Os08g22354	0.0036	−0.2196
	LOC_Os01g68320	0	−0.5106	LOC_Os05g08360	0.0041	0.3726
	LOC_Os03g50090	0	0.5609	LOC_Os02g52140	0.0046	−0.1969
	LOC_Os07g27300	0	0.5017	LOC_Os03g27030	0.0053	0.1488
	LOC_Os09g02400	0	−0.5812	LOC_Os05g08600	0.0071	0.1314
	LOC_Os03g49430	0	−0.2845	LOC_Os07g05050	0.0076	−0.2722
	LOC_Os03g22380	0	−0.1009	LOC_Os10g17454	0.0083	0.2608
	LOC_Os02g03040	0	−0.1717	LOC_Os01g69050	0.0085	−0.1132
	LOC_Os05g30140	0	−0.3902	LOC_Os02g05310	0.0101	−0.1912
	LOC_Os03g61990	0	0.2765	LOC_Os02g14780	0.0135	−0.3220
	LOC_Os07g47630	0.0001	0.2415	LOC_Os08g37960	0.0150	−0.1082
	LOC_Os06g49740	0.0001	0.1711	LOC_Os09g21520	0.0219	−0.1477
	LOC_Os05g48960	0.0004	−0.1464	LOC_Os09g33460	0.0269	−0.1503
	LOC_Os08g06344	0.0007	0.2430	LOC_Os07g07490	0.0306	−0.4725
	LOC_Os06g08840	0.0010	−0.2219	LOC_Os08g02130	0.0316	−0.1881
	LOC_Os02g39720	0.0011	−0.2701	LOC_Os04g58780	0.0317	−0.3272
	LOC_Os02g15310	0.0011	−0.3624	LOC_Os02g54770	0.0322	0.1542
	LOC_Os01g56520	0.0016	0.2188	LOC_Os07g31340	0.0397	−0.1910
	LOC_Os04g39060	0.0017	0.2188	LOC_Os02g31290	0.0402	−0.1802
	LOC_Os07g43050	0.0024	0.3328	LOC_Os04g39260	0.0431	−0.4523
	LOC_Os08g38410	0.0024	−0.1925	LOC_Os01g03060	0.0434	0.2008
	LOC_Os12g36740	0.0028	0.3393			

^1^ Values below 0.0001 are shown as 0. Each row has the lowest value among experiments 1–5 and where change in percentage spliced (ΔPS) was below 0.1. ^2^ ΔPS associated with the *p*-value.

## Data Availability

Datasets for this research are included in [14,19,20,22,62]. The script used to carry out the data mining analysis is available at https://github.com/charbavito/pub_project_rice_data_mining/blob/master/FTQ_script_v0.1.py. (accessed on 8 April 2021).

## Supplementary Materials

The following are available online at https://www.mdpi.com/article/10.3390/plants10081647/s1, Figure S1: Distribution of the number of genes (y-axis) per number of transcripts per gene (x-axis) before (red) and after (blue) filtering low expressed transcripts and genes using the RNA-seq data extracted from Luo et al. [62]; Figure S2: Venn diagram showing the overlap of expressed genes from each experiment analysed here; Figure S3: Number of up- and down-regulated genes; Figure S4: GO analysis of DAS genes identified in Experiment 3; Figure S5: Heat-induced changes in transcript expression of two DAS genes; Table S1: Sequencing information for each study analysed here; Table S2: Meta-data table containing experimental design information of 345 RNA-seq datasets analysed here; Table S3: DE and DAS genes IDs and functions, organised by experiment; Table S4: (a) Known heat-response genes, (b) novel heat-response DE and DAS genes, (c) novel DE-only heat shock proteins, transcription factors, epigenetic proteins and splicing-related factors.
