# Supplementary material for: Reading between the Lines: RNA-seq Data Mining Reveals the Alternative Message of the Rice Leaf Transcriptome in Response to Heat Stress
Source: Plants (Basel). 2021 Aug 11;10(8):1647. doi: 10.3390/plants10081647 (PMC8400768; doi:10.3390/plants10081647)
Supplement: Supplementary file 1 [file plants-10-01647-s001.zip › Supp_files/Supplementary File 1.pdf]

Supplementary File 1

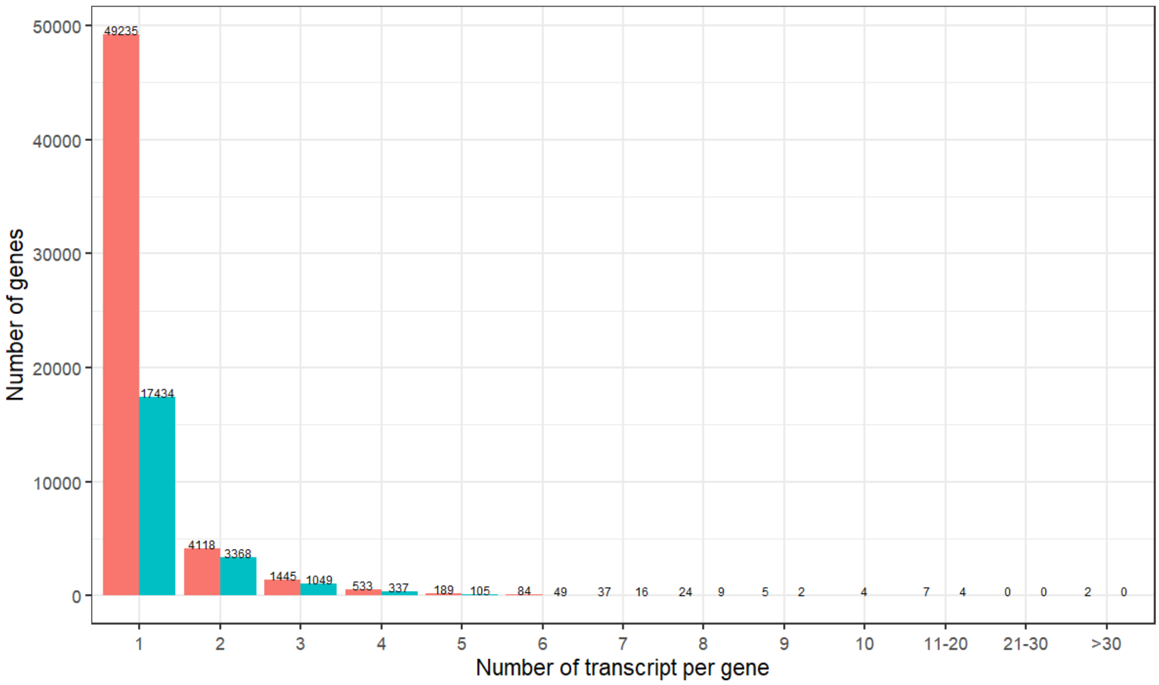

Figure S1. Distribution of the number of genes (y-axis) per number of transcripts per gene (x-axis) before (red) and after (blue) filtering low expressed transcripts and genes using the RNA-seq data extracted from Luo et al. [60] (Experiment 3).

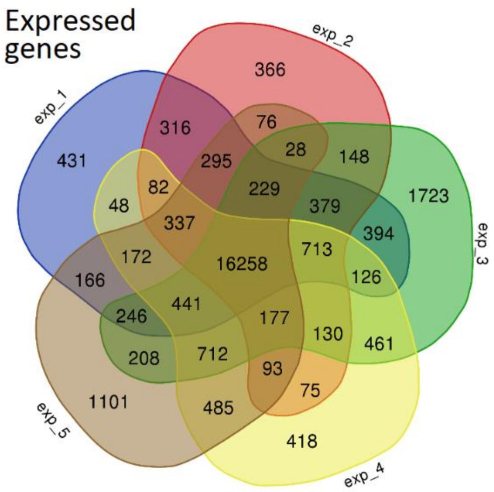

Figure S2. Venn diagram showing the overlap of expressed genes from each experiment analysed here.

	down-regulated	up-regulated
exp_1	472	68
exp_2	62	60
exp_3	6228	6422
exp_4	472	621

(**a**)

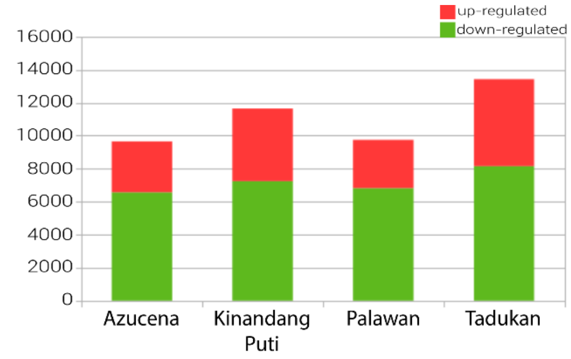

(**b**)

Figure S3. Number of up- and down-regulated genes. **(a)** Data from experiments 1, 2, 3 and 4. **(b)** Data from each rice cultivar analysed in experiment 5. y-axis = number of genes.

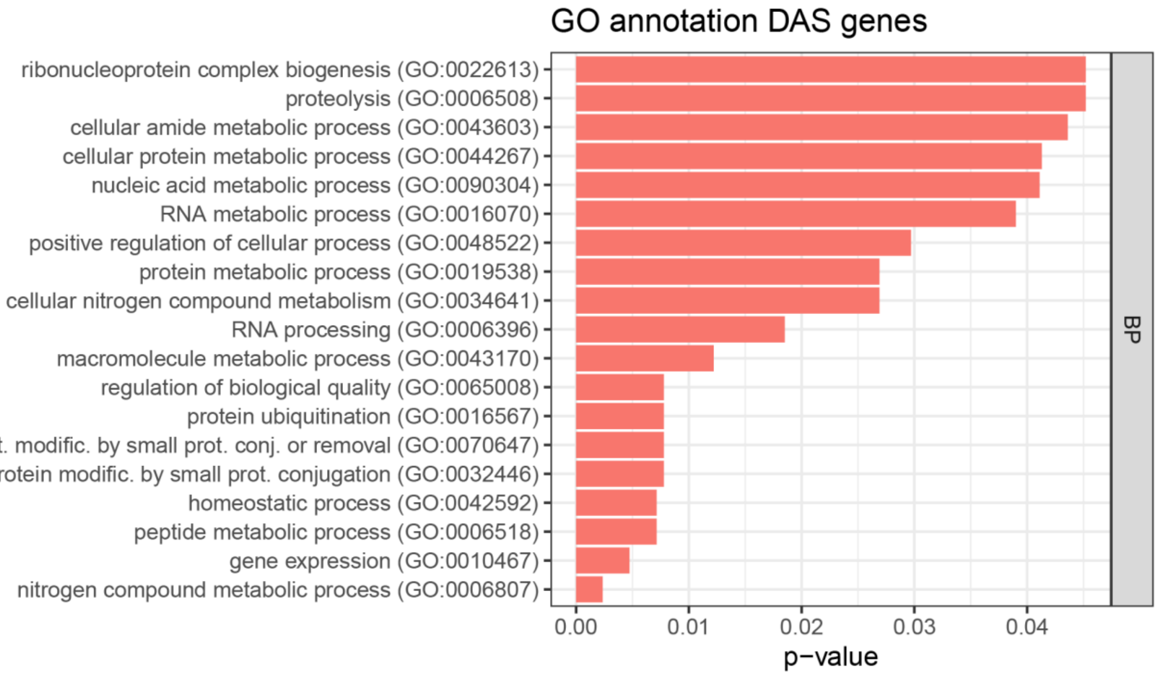

Figure S4. GO analysis of DAS genes identified in Experiment 3. The GO statistical overrepresentation test was carried out in Panther (http://pantherdb.org/) with the annotation set 'GO biological process complete' (BP). The reference list contained 4,349 expressed, multi-transcript (more than one isoform) and non-DAS genes from Experiment 3. The significantly enriched terms and associated *p*-values >0.5 were uploaded to 3D RNA-seq App [65] that generated the plot above.

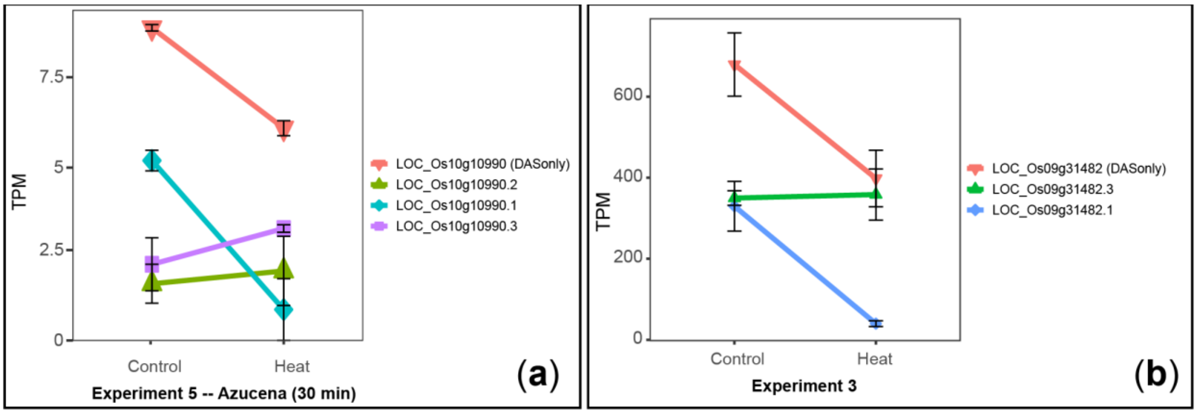

Figure S5. Heat-induced changes in transcript expression of two DAS genes. (**a**) LOC_Os10g10990. (**b**) *OsC3H60* (LOC_Os09g31482). Error bars: SEM.

Table S1. Sequencing information for each study analysed here.

Experiment	SRA Study	Number of datasets extracted	Average read length	Average number of reads	Biological replicates
1	SRP071314	24	100 bp	37M	2
2	SRP101342	4	50 bp	32M	2
3	SRP190858	6	297 bp	25M	3
4	SRP110860	4	150 bp	37M	2
5	SRP065945	307	102 bp	40M	2
